# The Relationship between Neuroticism, Hopelessness, and Depression in Older Korean Immigrants

**DOI:** 10.1371/journal.pone.0145520

**Published:** 2016-01-04

**Authors:** Bum Jung Kim, Kristen Linton, Sean Cho, Jung-Hwa Ha

**Affiliations:** 1 Myron B. Thompson School of Social Work, University of Hawaii at Manoa, Honolulu, HI, United States of America; 2 Health Sciences Program, California State University at Channel Islands, Camarillo, CA, United States of America; 3 Department of Social Welfare, Seoul National University, Seoul, South Korea; Leibniz Institute for Neurobiology, GERMANY

## Abstract

**Purpose:**

This study aimed to investigate the relationship between neuroticism, hopelessness, and depression among older Korean immigrants. To extend this line of research, this study aimed to examine the effects of neuroticism and hopelessness in predicting depression among older Korean immigrants.

**Methods:**

Data for this study came from a survey of 220 first generation Korean immigrants aged 65 years or older in Los Angeles County in 2012. Data were collected by face-to-face interviews with trained social workers using a structured questionnaire translated into Korean. All interviews were conducted in Korean. The neuroticism sub-scale of the Eysenck Personality Questionnaire was used to assess neuroticism (EPQN). Hopelessness was measured by the Beck Hopelessness Scale (BHS). Depression was measured by the 20-item Center of Epidemiological Studies Depression (CES-D) scale.

**Results:**

The study found that age (*β* = .26, *p*< .01), gender (*β* = -.13, *p*< .01), income (*β* = -.13, *p*< .01), neuroticism (*β* = .51, *p*< .01), and hopelessness (*β* = .15, *p*< .01) were significant predictors of depression.

**Conclusion:**

The study provides preventive strategies that would help in the development of depression-reduction services or programs for the population, especially for those living with neuroticism and hopelessness.

## Introduction

While 15–20% of the general population of older Americans experience depression, recent studies of older Korean immigrants found that between 24 and 30.3% experience depression [1, 2]. This shows that older Korean immigrants experience the highest rates of depression than the general population in the U.S. It should also be noted that older Koreans have the highest rates of suicide among all older ethnic groups in the U.S., which is often a consequence of depression [3]. Thus, it is essential to identify factors associated with depression among older Koreans to prevent and alleviate depression. Neuroticism, hopelessness, and other demographic characteristics have also been associated with depression among older adults in the general U.S. population, yet there is a lack of studies that assess their effects among older Korean immigrants [4].

Neuroticism is a personality trait characterized by worry, moodiness, and nervousness [5]. Higher levels of neuroticism have been positively associated with depressive symptoms among the general population of older adults in the U.S. [4, 6, 7]. Hayward and colleagues [4] found that decreasing neuroticism can result in an improvement of depression in their study of 112 older adults with major depression.

While the authors did not find a study specifically assessing the effects of neuroticism on depression among older Korean immigrants, research has found that Koreans experience higher levels of neuroticism than Americans [8, 9]. Thus, Korean American immigrants may also have higher levels of neuroticism. The higher prevalence of neuroticism may be associated with an aspect of Korean culture. Ka [10] explains that Koreans experience a “culture-bound emotion” (p. 222) called *jeong-han*, a negative affect related to neurotic sufferings. *Jeong-han* is a combination of *jeong* (affection) and *han* (hatred) and Koreans have a strong desire for *jeong*. This is experienced at a young age in which Koreans experience an intense desire to please their parents. This involves focusing more attention to their parent’s emotions than their own, resulting in self-neglect for their own emotions. Attempting to please parents among Korean children and adolescents involves a tremendous amount of focus which could result in neuroses. This desire for *jeong* is coupled with *han*, which includes pain, bitterness, and resentment due to personal tragedies, resulting in *jeong-han*. While a Korean American child may develop this attribute early on, it may remain a part of their personality throughout their lifetime [10].

The experience of neuroticism may change as Korean Americans experience acculturation since Korean values of *jeong* may decrease as they assimilate into American culture. Supporting this notion is research that has found that acculturation, measured by years since immigration and the use of the English language, has been negatively associated with depression and suicidal ideation among older Korean Americans [11, 12, 13].

It should also be noted that the experience of *han* is similar to hopelessness. Since Koreans have a strong desire to please their parents and though it may not always be successful, it may result in a negative view of the future and belief that nothing will turn out right, which is in essence, the definition of hopelessness. Hopelessness is a form of isolation which can strongly affect the risk of depression in individuals; thus, hopelessness is a determinant and component of depression. Mui and Kang [14] found that 40% of older Asian Americans including older Koreans experienced feelings of hopelessness. Moreover, studies have demonstrated that the construct of hopelessness is among the core features of depression [15, 16, 17].

In summary, older Korean immigrants are more likely than older Americans to experience depression [1, 2]. Neuroticism, or *jeong-han*, may be contributing to Korean Americans’ higher likelihood to experience depression [8, 9]. Hopelessness may also be a contributor to their depression [13, 14]. With this rationale, the study aimed to investigate the relationship between neuroticism, hopelessness, and depression among older Korean immigrants.

## Methods

### Data

Using a cross-sectional design, this study used data from a survey of 220 older Korean immigrants aged 65 years or older in Los Angeles County in 2012. Based on convenience sampling, the research team identified places that older Korean immigrants were presented, placed, and resided. The study sample was recruited from various settings, such as local Korean churches, senior plazas, adult day health care centers, and senior housing. The team contacted the program directors or managers of those places and provided the overall picture of the study in order to receive consent of agreement for participation. Eligible persons received information on the study’s purpose, survey questions, potential risks or harms, confidentiality, and anonymity. Inclusion criteria included: first generation immigrants of Koreans in origin, aged 65 and over, and living in Los Angeles County. The Human Subjects Protection Committee of the University approved this study. In addition, signed consent forms were obtained from all study participants. The response rate was 86%. Structured questionnaires were completed by face-to-face interviews with trained bilingual social workers. The questionnaire was translated into Korean using a back translation method. For accuracy and verification purposes, the Korean version was reviewed by a group of older Koreans to ensure that the questionnaire was congruent with the written Korean language and easily comprehensible. All interviews were conducted in Korean and upon completion, each participant received $20 as compensation.

### Measures

#### Neuroticism (EPQN)

The neuroticism sub-scale of the Eysenck Personality Questionnaire was used to assess neuroticism (EPQN). The EPQN contains 26 items scored according to a yes-no format. Neuroticism referred to a broad dimension of traits characterized by the tendency to experience negative emotion, including fearfulness, irritability, low self-esteem, social anxiety, and helplessness [18]. This study utilized the Korean version of the EPQN translated by Eysenck and Lee [19] with higher scores indicating more neuroticism. Previous work using the EPQN scale for older Korean immigrants also yielded acceptable reliability and validity levels [20, 21]. Internal consistency of the scale in this study was .94.

#### Hopelessness (BHS)

Hopelessness was measured using the Beck Hopelessness Scale [22]. The 20-item BHS included questions about three major aspects of hopelessness: feelings about the future, loss of motivation, and expectations. Each question required a true or false answer. The higher score implied greater hopelessness. Previous work using the BHS scale with older Korean populations also yielded acceptable reliability and validity levels [5, 21]. The internal reliability for the BHS in this study was .91.

#### Depression (CES-D)

Depression was measured by the 20-item Center of Epidemiological Studies Depression (CES-D) scale [23]. The CES-D contained questions about psychological, physical, and emotional symptoms, which emerge when people are emotionally distressed. Such symptoms include loss of appetite, sleep restlessness, and feeling lonely, satisfaction with life, feeling isolated, sad, or blue. Responses for these items were elicited along a four-point continuum frequency, which ranged from rarely or none (0) to most of the time (3). Higher scores implied greater depression. Previous work using the CES-D scale with older Korean population also yielded acceptable reliability and validity levels [18, 13]. Cronbach’s alpha for this 20-item scale for this sample was .93.

#### Demographic and cultural variables

Demographic variables in the study includes the demographic and socioeconomic characteristics: age (in years), gender (male/female), marital status (single/married), income, education (in years). In addition, other variables, such as: the length of stay in the U.S. and acculturation, were included. Acculturation was measured by a brief-version of the Suinn-Lew Asian Self Identify Acculturation scale. This brief version [24, 25] consists of five items (language spoken, language preferred, language read, childhood friends, and self-assessed level of acculturation). The score of each item ranges from 1 (Korean-culture oriented) to 5 (American-culture oriented), and the total score is the mean of the five-item scores. This acculturation scale in the present study has high reliability (Cronbach’s alpha = .92).

#### Analysis

Descriptive statistics were used to explain the basic characteristics of the study variables. Also, Pearson’s correlation analysis was used to investigate the bivariate relationship between independent (age, income, education, length of stay in the U.S., acculturation, neuroticism, and hopelessness) and dependent variables (depression) in order to check the strength of relationships and multicollinearity. An independent sample *t*-test for gender and marital status was done to compare means. Finally, a multiple regression analysis was conducted to examine the effects of major independent variables (neuroticism & hopelessness) on depression, while controlling for the socio-cultural variables. The study created two Models introducing independent variables in the following steps: a) Model 1 includes socio-cultural variables (age, gender, marital status, income, education, length of stay in the U.S., acculturation) as control factors. b) Model 2 included key independent variables such as neuroticism and hopelessness. STATA 13 [15] was used for all data analyses.

## Results

### Descriptive Statistics

Descriptive statistics are presented for the measures used (Table 1). Ages ranged from 65 to 92, with a mean age of 74.70 (*SD* = 7.78). Approximately 65% were female and 48% were married. The average monthly income was $1,381 with a range from $0 to $5,000 and about an average of 10.6 years of education obtained. For the length of stay, study participants averaged 9.6 years (*SD* = 4.07) in the U.S. while the average score for acculturation was 2.58 (*SD* = 2.56, range = 0–9). The average score for neuroticism in this sample was 5.18 (*SD* = 6.27). For hopelessness, the respondents showed an average of 7 (*SD* = 4.46) and depression averaged 12.5 (*SD* = 8.68).

**Table 1:** Descriptive Statistics for Raw Scores of Measures (*N* = 220).

Variable	*n*	*%*
Age (in years)		
Range	65–92	
Mean (*SD*)	74.70 (7.78)	
Gender		
Female	145	65.91
Male	75	34.09
Marital Status		
Not married	115	52.27
Married	105	47.73
Income (in dollar)		
Range	0–5000	
Mean (*SD*)	1381.53 (1065.21)	
Education (in years)		
Range	0–20	
Mean (*SD*)	10.65 (5.08)	
Length of Stay (in years)		
Range	0–30	
Mean (*SD*)	9.58 (4.07)	
Acculturation		
Range	0–9	
Mean *(SD*)	2.58 (2.56)	
Neuroticism		
Range	0–29	
Mean (*SD*)	5.18 (6.27)	
Hopelessness		
Range	0–18	
Mean (*SD*)	7.00 (4.46)	
Depression Range	0–36	
Mean (*SD*)	12.5 (8.68)	

Gender: Female = 0, Male = 1, Marital Status: Single = 0, Married = 1,

### Bivariate Correlations

In order to determine the degree of multicollinearity among the study variables, the bivariate correlation coefficients using *Pearson’s r* were examined. The absence of multicollinearity was confirmed (VIF values: acculturation = 2.53, age = 2.18, hopelessness = 2.09, education = 2.02, income = 2.01, neuroticism = 1.96, length of stay = 1.39)). Older Korean immigrants who had higher levels of depression were more likely to be older (r = .38, p < .01), have lower income (r = -.38, p < .01), lower levels of education (r = -.32, p < .01), acculturation (r = -.45, p < .01), higher levels of neuroticism (r = -.54, p < .01) and hopelessness (r = .45, p < .01) (Table 2). Also, Table 3 shows that women (74.7%) were more likely to report higher depression than men (25.2%). Single adults (71.4%) were more likely to report higher depression when compared to the married adults (28.5%). The study found significant differences between gender and marital groups in reporting depression (Table 3).

**Table 2:** Correlations among Study Variables for Older Korean Immigrants (r scores, N = 220).

	1	2	3	4	5	6	7	8	9	10
1. Age	-									
2. Income	-.65**									
3. Education	-.29**	.38**								
4. Length of Stay	.14*	-.06	-.01							
5. Acculturation	-.45**	.38**	.54**	.32**						
6. Neuroticism	.20**	-.22**	-.22**	-.17*	-.30**					
7. Hopelessness	.23**	-.35**	-.50**	-.05	-.32**	.53**				
8. Depression	.38**	-.38**	-.32**	-.11	-.45**	.54**	.45**			-

* p < .05.

** p < .01.

Gender: Female = 0, Male = 1, Marital Status: Single = 0, Married = 1

**Table 3:** T-test (Gender & Marital status & Depression) for Older Korean Immigrants.

Characteristics		Low Depression (N = 129)	High Depression (N = 91)	Significance
	Gender			
	Men	40.31	25.27	*P < .05
	Women	59.69	74.73	
	Marital status			
	Married Single	61.24	28.57	**P < .01
		38.76	71.43	

*p < .05,

**p < .01

### Predictive Model of Depression

Using multiple regression analysis, three models (Table 4) were completed. Model 1 includes demographic and cultural variables which were entered simultaneously on the first step. The first model explained 20% of the total variance. Age (*β* = .32, *p*< .01), marital status (*β* = -.19, *p*< .01), income (*β* = -.16, *p* < .05), and length of stay (*β* = -.04, *p* < .05) significantly predicted depression. Older Korean immigrants who were older, single, and had less income were likely to have higher levels of depression. The second model explained that 52% of the total variance with indication of age (*β* = .26, *p*< .01), gender (*β* = -.13, *p*< .01), income (*β* = -.13, *p*< .01), neuroticism (*β* = .51, *p*< .01), and hopelessness (*β* = .15, *p*< .01) were significant predictors of depression.

**Table 4:** Multiple Regression Models of Depression among Older Korean Immigrants.

Variables	Model 1		Model 2	
	Beta	T value	Beta	T value
Age	.32	4.16**	.26	5.01**
Gender	-.08	-1.27	-.13	-3.05**
Marital Status	-.19	-2.85**	.07	1.40
Income	-.16	-2.21*	-.13	-2.72**
Education	-.09	-1.33	.05	1.08
Length of Stay	-12	-2.06	-.03	-.71
Acculturation	-.04	-.55	-.09	-1.65
Neuroticism			.51	10.11**
Hopelessness			.15	3.14**
R2	.20		.52		
Adjusted R2	.18		.51		
F	20.80**		40.86**		

* p < .05.

** p < .01.

## Discussion

The current study aimed to examine the effects of neuroticism and hopelessness in predicting depression among older Korean immigrants. Given the fact that depression is a prevalent mental health issue for this population, the study’s findings described here broaden our comprehension of the risk factors related to depression and suggest the practice and policy implications for developing and implementing effective preventive services. In all, neuroticism and hopelessness were found to be significantly associated with depression. Additionally, age, gender, and marital status were found to have significant effects on depression.

### Neuroticism and Depression

As noted, neuroticism showed the strongest direct effect on depression in this study’s findings. Older Korean immigrants with severe neuroticism are more likely to experience higher levels of depression. This result is consistent with previous studies [26, 6, 7]. It is defined that neuroticism is a fundamental personality trait characterized by anxiety, worry, and excessive stress [5]. A number of causes triggering neuroticism in older Korean immigrants can be considered.

First, it can be related to a number of cultural obstacles such as: language barriers and culture shock, especially throughout immigration process. A substantial number of older Koreans migrated to the U.S. during an era of political and economic hardships in South Korea while holding on to the American dream with an expectation of better lives [27, 28]. However, they may have had difficulties after their immigration due to the difficulties in adjustment, language proficiency, and discrimination [29, 30]. This hardship continues to exist even during their old age as most of them are still struggling with social adjustment relevant to cultural assimilation and English abilities [31, 32]. Accordingly, this makes it difficult for them to have access to social services including medical care, so they may face more challenges in managing their health and daily lives [33] as compared to other groups. All these situational difficulties place them in severely stressful conditions which is an indicator of neuroticism. Secondly, there is a lack of social supports and networks among older Korean immigrants. Since they have been separated from most of their relatives, acquaintances, and friends including childhood friends in South Korea upon immigration; they may not have adequate social supports to express and vent their emotions and distress too. Even the task of making new close-friends become a challenge due to their busy and difficult lives as well as the fear of possible fraud, jealousy, competition, and discrimination among the others in their community [34]. Thus, by having fewer opportunities to release and vent their stress using social networks and supports, they experience neuroticism.

It is clear that older Korean immigrants with higher levels of stress tend to suffer from neuroticism and it ultimately effects depression [5, 11]. As for preventive methods regarding neuroticism; minimizing language barriers should be emphasized by providing language education at an educational or community setting as well as offer bilingual services for social services to assist them in receiving social benefits. Additionally, it is recommended to use religious and social groups that offer programs for extending social networks in order to boost social interaction with others.

### Hopelessness and Depression

This study described that hopelessness is positively and significantly associated with depression. These findings are consistent with previous literature on older adults including older Korean immigrants [35, 36]. There are several reasons why hopelessness is prevalent for the study population. The most critical reason is related to their economic insecurity with a high poverty rate. The poverty rate of older Koreans is 23% which is higher than elder Whites (7%) and other racial groups [37]. The higher poverty rates are derived from being employed in low-skilled jobs with lower salaries and income [38, 39]. Upon retirement, they may have tried to find jobs which helps in earning more income however, it may be very difficult, not only due to their old age, but also language barriers and physical limitations which potential employers tend to avoid [34]. Also, it is well-known that Korean Americans have a very strong desire for their children to have an upstanding education; in order to help their children become successful. However, with financial hardships, they may experience extreme guilt and feel hopeless since they are not able to provide enough educational opportunities [40]. Another reason is their living arrangements. In addition to the increased worries about various economic challenges caused by the financial crisis and having decreased savings, in comparison to others, older Korean immigrants are more likely to live alone or with only their spouse [21, 41]. There is empirical evidence which shows that older adults living alone tend to feel lonely and hopeless which results in higher rates of depression and suicide [40, 41]. In this perspective, higher levels of hopelessness derived from economic hardships and family structural changes can trigger excessive stress which can result in experiencing depressive symptoms.

In terms of preventive strategies to reduce feelings of hopelessness among older Korean immigrants, stakeholders especially, community leaders and policy members, need to implement policies or services for employment extension in order to assist in increasing their income. Additionally, there should be considerations in developing countermeasures for their children's education such as student loan provisions targeting immigrant families with reasonable interest rates and high quality educational services for children with immigrant backgrounds. Moreover, both increasing the availability of senior housing and activating community engagement programs would be beneficial to help connect them with peers and help to release their hopeless emotions. Finally, it is recommended using the BHS scale to assist in identifying potential older Koreans risk of suicide, since hopelessness is strongly associated with depression and suicide. Healthcare professionals need to conduct a suicide risk assessment using the BHS since the BHS is strongly related in identifying suicide risk [28].

## Study Limitations and Conclusion

Some limitations of this study will be addressed. Due to the limited number of the study participants, generalizability of the study for other Koreans in different settings or other Asian groups is limited. Future data collection should be implemented in various locations or places around the nation. Another limitation is related to the study design. In using a cross-sectional survey design, the study cannot confirm the causal relationship among the study variables. A longitudinal-based design can be suggested for a more rigorous analysis. In addition, this study did not include stress and its various types as a study variable and its effects on neuroticism and hopelessness. Finally, since the structure of the hopelessness (BHS) scale may differ across clinical and nonclinical groups [33], it is recommended to conduct future research studies by separating those groups.

Despite these challenges, this study provides solid practical evidence for the literature in terms of the associations among the key factors of depression, and as such, provides preventive strategies that would help in the development of depression-reduction services or programs for the population, especially for those with neuroticism and hopelessness in their lives.

## Supporting Information

S1 Data Data File. (XLSX)

## Abbreviations

EPQN: Eysenck Personality Questionnaire—Neuroticism
BHS: Beck Hopelessness Scale
CES-D: Center of Epidemiological Studies Depression scale
